# Supplementary material for: Cutoff Points of Waist Circumference for Predicting Incident Non-Alcoholic Fatty Liver Disease in Middle-Aged and Older Korean Adults
Source: Nutrients. 2022 Jul 21;14(14):2994. doi: 10.3390/nu14142994 (PMC9323785; doi:10.3390/nu14142994)
Supplement: Supplementary file 1 [file nutrients-14-02994-s001.zip › nutrients-1805167-supplementary.pdf]

Supplementary Materials

Table S1. Baseline characteristic of the study population according to incident NAFLD.

Variables	Total (*n* = 5400)	No incident NAFLD (*n* = 3044)	incident NAFLD (*n* = 2356)	p-value
Age, years	51.7 ± 8.8	51.4 ± 9.0	52.0 ± 8.5	0.007
WC, cm	80.0 ± 8.1	77.6 ± 7.7	83.1 ± 7.4	<0.001
Body fat, %	26.3 ± 7.2	25.0 ± 7.1	28.0 ± 7.0	<0.001
BMI, kg/m^2	23.9 ± 2.9	23.0 ± 2.7	24.9 ± 2.7	<0.001
Total muscle mass, kg	42.0 ± 7.6	41.4 ± 7.3	42.9 ± 7.8	<0.001
Height-adjusted TMM, kg/m^2	16.5 ± 1.7	16.2 ± 1.6	16.8 ± 1.6	<0.001
BMI-adjusted TMM, m^2	1.8 ± 0.3	1.8 ± 0.3	1.7 ± 0.3	<0.001
Body weight, kg	60.5 ± 9.1	58.5 ± 8.6	63.1 ± 9.0	<0.001
Smoking status, *n* (%)				0.930
Non-smoker	3561 (66.8)	2019 (67.1)	1542 (66.4)	
Ex-smoker	683 (12.8)	380 (12.6)	303 (13.0)	
Intermittent smoker	114 (2.1)	65 (2.2)	49 (2.1)	
Every day smoker	973 (18.3)	543 (18.1)	430 (18.5)	
Physical activity, *n* (%)				0.290
Low (<7.5 METS-h/wk)	392 (7.6)	222 (7.6)	170 (7.5)	
Moderate (7.5–30 METS-h/wk)	3148 (60.6)	1802 (61.5)	1346 (59.5)	
High (>30 METS-h/wk)	1651 (31.8)	906 (30.9)	745 (33.0)	
Alcohol drinking, yes, *n* (%)	2305 (43.0)	1282 (42.5)	1023 (43.8)	0.335
Total energy intake, kcal/day	1937.1 ± 706.1	1930.2 ± 691.2	1946.0 ± 725.0	0.425
MBP, mmHg	94.0 ± 12.6	92.1 ± 12.5	96.3 ± 12.4	<0.001
FPG, mg/dl	82.6 ± 11.8	81.0 ± 8.4	84.6 ± 14.9	<0.001
TC, mg/dl	188.0 ± 33.6	185.0 ± 32.7	191.7 ± 34.3	<0.001
CRP	0.23 ± 0.60	0.2 ± 0.5	0.3 ± 0.7	0.001
ALT	22.1 ± 8.8	20.8 ± 8.0	23.8 ± 9.5	<0.001
AST	26.4 ± 6.9	26.1 ± 6.7	26.8 ± 7.1	<0.001

Data are presented as mean ± standard deviations for continuous variables and number (%) for categorical variables. Abbreviations: WC, waist circumference; BMI, body mass index; TMM, total skeletal muscle mass; MBP, mean blood pressure; FPG, fasting plasma glucose; TC, total cholesterol; CRP, C reactive protein; ALT, alanine aminotransferase; AST, aspartate aminotransferase; NAFLD, non-alcoholic fatty liver disease

Table S2. Harrell's concordance index and 95% confidence interval for incident non–alcoholic fatty liver diseases according to five body composition indices.

	Variables	Harrell's concordance index (95% CI)	Pairwise comparison *p*-value				
			WC	Body fat	BMI	TMM/Ht^2	TMM/BMI
**Total**	**WC**	0.68 (0.67–0.69)	reference				
	**Body fat**	0.59 (0.58–0.60)	<0.001	reference			
	**BMI**	0.66 (0.65–0.68)	0.001	<0.001	reference		
	**TMM/Ht^2**	0.60 (0.59–0.61)	<0.001	0.616	<0.001	reference	
	**TMM/BMI**	0.56 (0.55–0.57)	<0.001	<0.001	<0.001	0.001	reference
**Men**	**WC**	0.68 (0.66–0.70)	reference				
	**Body fat**	0.64 (0.62–0.66)	<0.001	reference			
	**BMI**	0.66 (0.64–0.68)	0.001	0.033	reference		
	**TMM/Ht^2**	0.61 (0.59–0.63)	<0.001	0.007	<0.001	reference	
	**TMM/BMI**	0.58 (0.56–0.60)	<0.001	<0.001	<0.001	0.033	reference
**Women**	**WC**	0.69 (0.67–0.70)	reference				
	**Body fat**	0.64 (0.63–0.66)	<0.001	reference			
	**BMI**	0.67 (0.66–0.68)	0.001	<0.001	reference		
	**TMM/Ht^2**	0.62 (0.61–0.64)	<0.001	0.070	<0.001	reference	
	**TMM/BMI**	0.62 (0.60–0.63)	<0.001	<0.001	<0.001	0.702	reference

Abbreviations: WC, waist circumference; BMI, body mass index; TMM, total skeletal muscle mass; CI, confidence interval.
